# Cross-sectional trend analysis of the NCHA II survey data on Canadian post-secondary student mental health and wellbeing from 2013 to 2019

**DOI:** 10.1186/s12889-021-10622-1

**Published:** 2021-03-25

**Authors:** Brooke Linden, Randall Boyes, Heather Stuart

**Affiliations:** 1 Health Services and Policy Research Institute, Queen’s University, Abramsky Hall, 21 Arch Street, Kingston, ON K7L 3N6 Canada; 2 Department of Public Health Sciences, Queen’s University, Abramsky Hall, 21 Arch Street, Kingston, ON K7L 3N6 Canada

**Keywords:** Mental health, Postsecondary, Stress, Mental illness, Help seeking

## Abstract

**Background:**

Canadian post-secondary students are considered to be at risk for chronic stress and languishing mental health, but there has been no longitudinal analysis of the available population-level data. The purpose of this study was to examine trends in the overall and sex-specific prevalence of self-reported stress, distress, mental illness, and help seeking behaviours among Canadian post-secondary students over the past several years.

**Methods:**

Using the 2013, 2016, and 2019 iterations of the National College Health Assessment II Canadian Reference data, we conducted a trend analysis for each variable of interest, stratified by sex. The significance and magnitude of the changes were modelled using cumulative linked ordinal regression models and log binomial regression models.

**Results:**

With few exceptions, we observed significant increases over time in the proportion of students reporting symptoms of psychological distress, mental illness diagnoses, and help seeking for mental health related challenges. Female students reported a higher level of stress than male students, with a statistically significant increase in the stress level reported by female students observed over time. In all cases, larger proportions of female students were observed compared to male students, with the proportion of female students who self-reported mental illness diagnoses nearly doubling that of males.

**Conclusions:**

Our analysis indicated that the proportion of students self-reporting mental health related challenges, including stress, psychological distress, and diagnosed mental illnesses increased between the 2013, 2016 and 2019 iterations of the NCHA II conducted among Canadian post-secondary students.

**Supplementary Information:**

The online version contains supplementary material available at 10.1186/s12889-021-10622-1.

## Background

Increasingly, post-secondary students are acknowledged as an at-risk group for poor mental health and the development of mental illnesses. Research has demonstrated that students report a high prevalence of stress [1, 2] and mental illnesses [3–5]. The 2012 cycle of the Canadian Community Health Survey revealed that young Canadians aged 15–24 years (capturing the majority of post-secondary students) were the most likely to report symptoms of a mental illness or an unmet need for mental health care [6, 7]. The 2016 Global Burden of Disease Study revealed mental illnesses to be among the top four leading causes of loss of disability-adjusted life-years (DALYs) in Canada [8].

Post-secondary students face a wide array of stressors, spanning the academic, financial, and social spheres, placing them at risk for languishing mental health, particularly if they lack effective coping strategies. Chronic stress is highly correlated with negative mental health outcomes and has been shown to have a substantial impact on students’ academic performance [9, 10]. Research suggests that male and female students experience mental health challenges differently, with females more likely to report symptoms consistent with anxiety and depression [11], and males more likely to experience issues related to perceived stigma and help seeking [12–14]. Flourishing mental health and emotional wellbeing are prerequisites for academic success, and mental health problems are associated with academic failure, dropout, addictions, and even suicide [15–17], underscoring the importance of monitoring students’ mental health.

Currently, there is no nationally coordinated effort to monitor mental health related data for post-secondary students, though one is currently in development at the University of British Columbia [18]. This is an important gap to be filled, given that the design and implementation of interventions to alleviate stress and promote mentally healthy campuses should be informed by the prevalence and correlates of languishing mental health. In the absence of a Canadian-made surveillance system, the National College Health Assessment Survey II (NCHA II), delivered by the American College Health Association, constitutes the most complete source of data on post-secondary student stress and mental health outcomes currently available. To date, the NCHA II has been conducted on three occasions among Canadian post-secondary students, producing mental health-related data for 2013, 2016, and 2019. To our knowledge, a longitudinal analysis of these data has not yet been conducted. Therefore, the primary purpose of this secondary analysis of cross-sectional data was to examine trends in the overall and sex-specific prevalence of self-reported stress, psychological distress, mental illness, and help seeking behaviours among Canadian post-secondary students from 2013 to 2019.

## Methods

Data were collected cross-sectionally from students at participating Canadian post-secondary institutions in 2013 (*n* = 38,171 student responses), 2016 (*n* = 43,780), and 2019 (*n* = 55,284). Mean response rates^1^ for each year ranged from 19 to 20% [19–21]. The number of post-secondary institutions that participated in each year were 32, 41, and 58 in 2013, 2016, and 2019, respectively. The NCHA II consists of over 300 questions relating to the overall health of post-secondary students. While the survey is primarily concerned with physical health indicators, a number of questions pertain to students’ emotional and mental health. The NCHA II is administered confidentially through Qualtrics by the ACHA. Each participating institution provides the ACHA with a fee for participation, a letter of information and informed consent, a subject line for the invitation, a copy of the institution’s research ethics board approval, and a sample of students’ e-mail addresses. The sampling method and sample size used is at the discretion of each participating institution. After the initial distribution of the survey, non-responders are sent up to three reminder e-mails. A total of 32 institutions participated in 2013, 41 in 2016, and 58 in 2019.

### Measures

#### Demographics

To determine the characteristics of the samples used we analyzed the following demographic characteristics of participants: age, sex, year in school, enrollment status, and estimated grade point average (GPA). All demographic variables were categorical in nature, and recoded for ease of presentation (e.g., age group).

#### Stress and psychological distress

Participants were asked to rate their general stress level over the past 12 months on a five-point ordinal scale ranging from “no stress” to “tremendous stress.” Participants were also asked to indicate the frequency with which they had felt symptoms of psychological distress within the past 12 months by responding to eleven items. Response categories were coded as “yes” or “no.”

#### Mental illness

Participants were asked whether they had been diagnosed by a mental health professional for a number of mental illnesses within the past 12 months. Response categories were coded as “yes” or “no.” An additional question assessed whether participants had ever been diagnosed with depression. Responses were coded as “yes” or “no.”

#### Help seeking

To assess help seeking behaviours, participants were asked whether they had ever received psychological or mental health services from a variety of providers. Responses were coded as “yes” or “no.” Next, participants were asked whether they would seek help in the future for a mental health problem, should the need arise. Responses were categorized as “yes” or “no.”

### Analysis

We conducted a secondary analysis of the cross-sectional data. Descriptive statistics were calculated for all demographic variables at each time point. We used a cumulative link mixed effects model (ordinal variable) and log binomial regression models (dichotomous variables) to determine whether the distribution of responses (ordinal) or prevalence estimates (dichotomous) had changed significantly over time using risk ratios and 95% confidence intervals. In all models, school was entered as a random effect to control for possible clustering. In order to compare the rate of change in female and male students, we included biological sex as an interaction term in the models. Results were then visually displayed for each variable of interest using appropriate graphs. Relative measures indicating the multiplicative change per year were calculated using these models. For example, an RR of 1.10 should be interpreted to mean that a prevalence of 20% in 2013 changed to 22% in 2014, 24.2% in 2015, and so on. We also calculated a relative measure of the difference in prevalence between males and females in the final year of data availability. Each model was developed using a complete case analysis approach to missing data; any student who had complete information for the variables relevant to an individual model was included in the analysis for the purposes of that model. Final analytical sample sizes for each model are reported in the supplemental table found in Additional file 2.

## Results

Table 1 presents the demographic characteristics for each sample. In each year of data collection, the largest proportion of participants were full-time students, female, and reported a GPA in the B range. The majority of students were between 18 and 24 years of age, and studied at the undergraduate level. The amount of missing data for each outcome was low; complete case analysis resulted in the inclusion of at least 97% of students in each model.

**Table 1:** Demographic Characteristics of Samples 2013–2019

		2013		2016		2019	
		Freq. (n)	Percent (%)	Freq. (n)	Percent (%)	Freq. (n)	Percent (%)
**Sex**	Female	22,995	68.6	30,373	70.0	38,127	69.5
	Male	10,519	31.4	13,035	30.0	16,760	30.5
**Age**	18–20 years	13,412	39.9	17,418	40.3	21,480	39.4
	21–24 years	12,939	38.5	16,187	37.4	19,050	34.9
	25–29 years	4149	12.3	5398	12.5	7519	13.8
	30+ years	3131	9.3	4241	9.8	6481	11.9
**Year in School**	1st year UG	6851	20.5	9949	23.0	14,355	26.3
	2nd year UG	6808	20.4	8843	20.4	12,538	22.9
	3rd year UG	6594	19.7	8040	18.6	8845	16.2
	4th year UG	5641	16.9	6331	14.6	6540	12.0
	5th year+ UG	2221	6.6	2858	6.6	2666	4.9
	Graduate/Professional	4834	14.5	6026	13.9	6873	12.6
	Not seeking a degree	152	0.5	439	1.0	1045	1.9
	Other	316	0.9	789	1.8	1805	3.3
**Enrollment**	Full-time	31,003	92.5	40,528	93.3	51,129	93.1
	Part-time	2227	6.6	2454	5.6	3237	5.9
	Other	278	0.8	478	1.1	552	1.0
**GPA**	A	11,296	34.5	15,799	37.1	21,312	39.7
	B	15,772	48.2	19,562	45.9	23,770	44.3
	C	5202	15.9	6619	15.5	7856	14.6
	D/F	479	1.5	626	1.5	748	1.4
**Total Sample**		**34,039**		**43,780**		**55,284**	

This file contains Table 1, which displays the demographic characteristics of the samples used in the presented analysis (please see indication for placement in body of manuscript)

### Stress

**Fig. 1:**
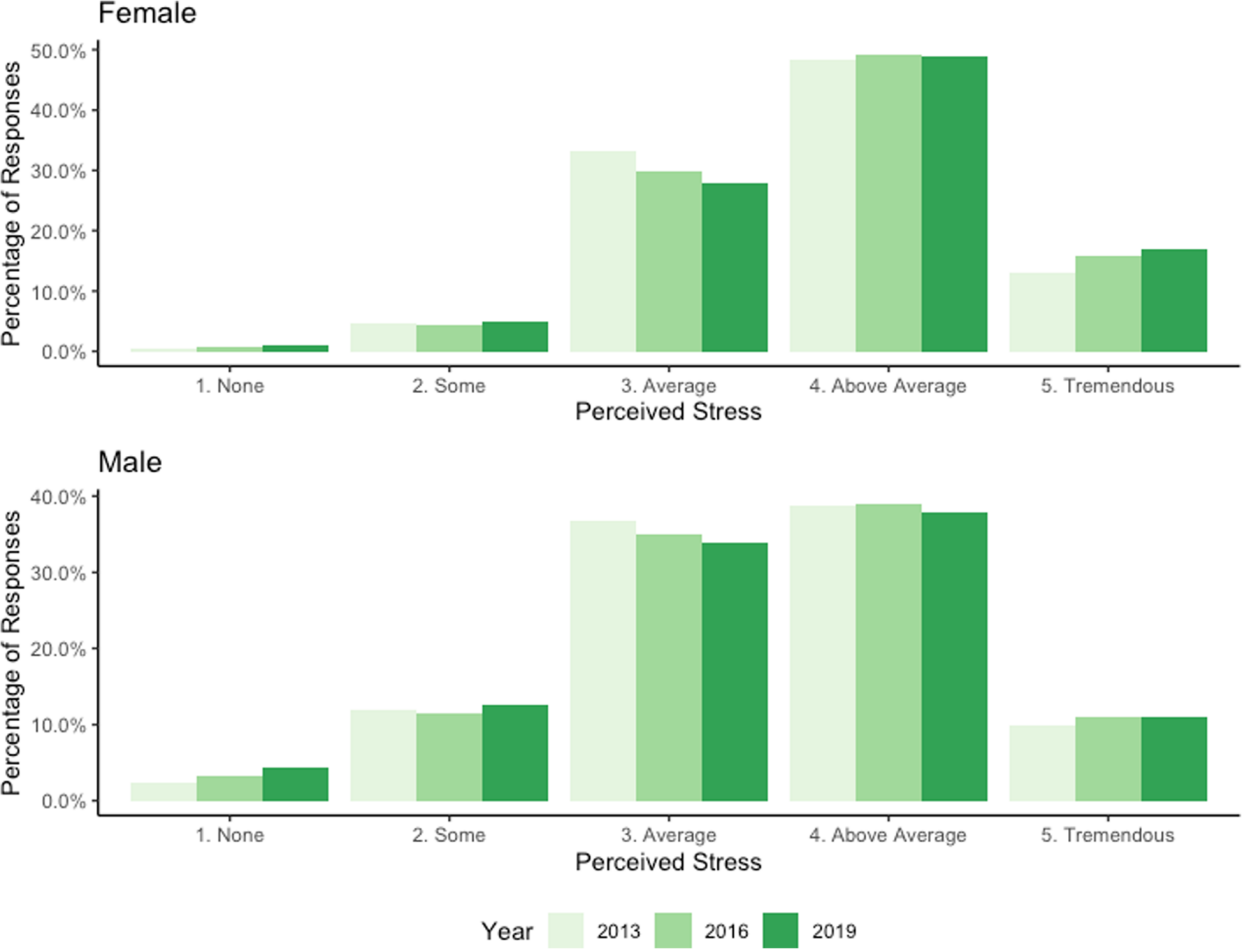
Proportion reporting past 12-month stress levels over time, by sex

Students were asked to rate the overall level of stress they had experienced within the past 12 months on a five-point ordinal scale ranging from ‘no stress’ to ‘tremendous stress’ (Fig. 1). We analyzed this variable as both a continuous variable and as an ordinal variable. Observing the continuous measure of stress, female students reported a higher average stress level than male students (*p* < 0.001), and we observed a statistically significant increase in the average stress level reported by female students over time (*p* < 0.001), but not in the average stress level reported by male students (*p* = 0.656). Observing ordinal responses, a larger proportion of females than males reported ‘above average’ or ‘tremendous stress,’ while a larger proportion of male students reported ‘no stress’ (Fig. 1).

### Psychological distress

**Fig. 2:**
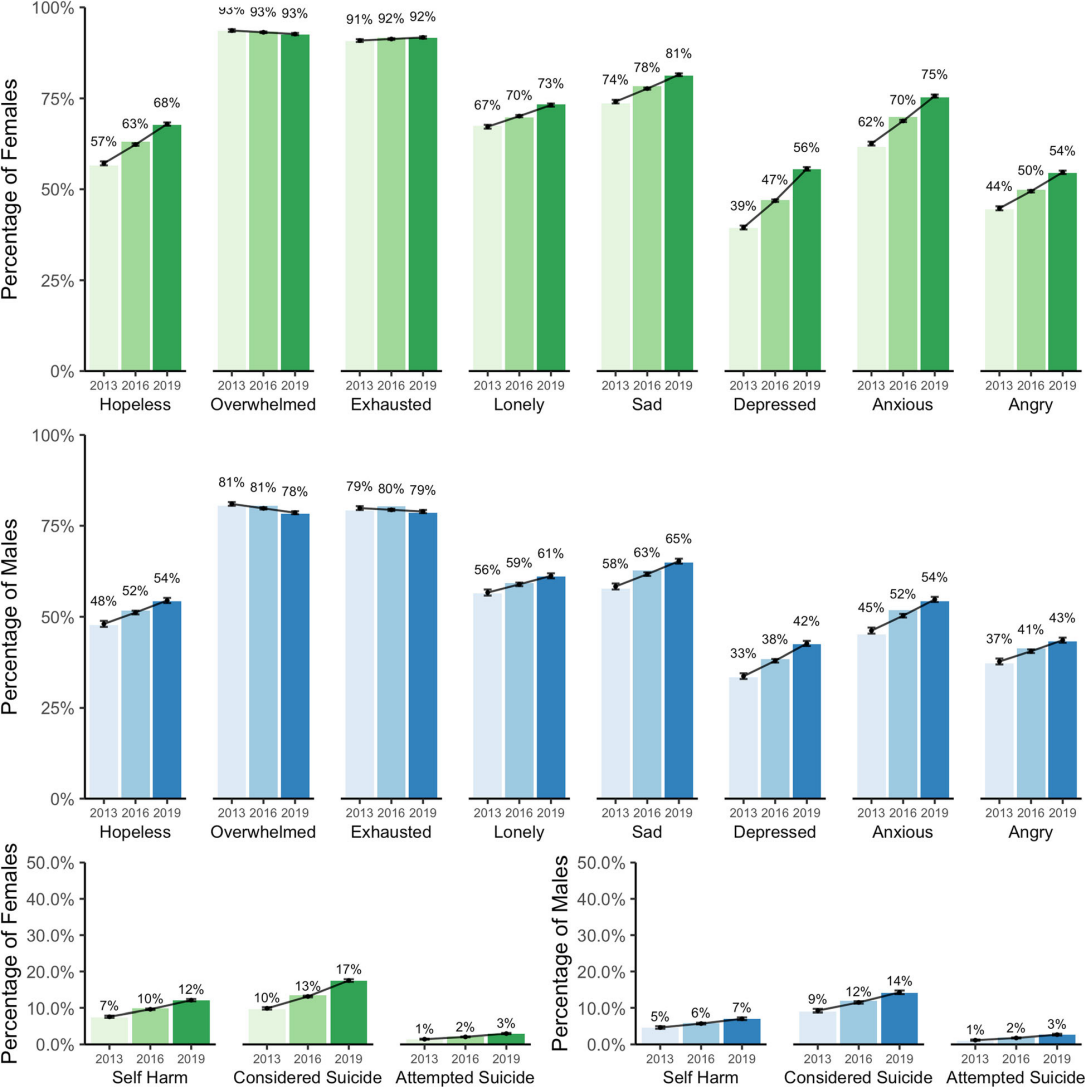
Proportion reporting symptoms of general (above) and extreme (below) psychological distress over time, by sex

Eleven items were used to evaluate symptoms of psychological distress within the past 12-month period (Fig. 2). The final three items reflect the most extreme manifestations of distress (self-injury, suicidal ideation, and suicide attempts). Significant increases were observed in the proportion of students reporting symptoms over time with few exceptions.

The proportion of students reporting feeling “overwhelmed” or “exhausted” remained stable over time for both males and females, while notable increases were observed for all other symptoms. Though the increases observed among self-harm, suicidal ideation, and suicide attempts were modest, they were statistically significant, and any increase in the proportion of students reporting these symptoms is concerning given their severity. The largest increases were observed for feeling “so depressed it was difficult to function” and feeling “overwhelming anxiety” for both males and females (see Additional file 1 for risk ratios and *p*-values). Notably, larger proportions of female students were observed compared to male students across all symptoms of psychological distress.

### Mental illnesses

**Fig. 3:**
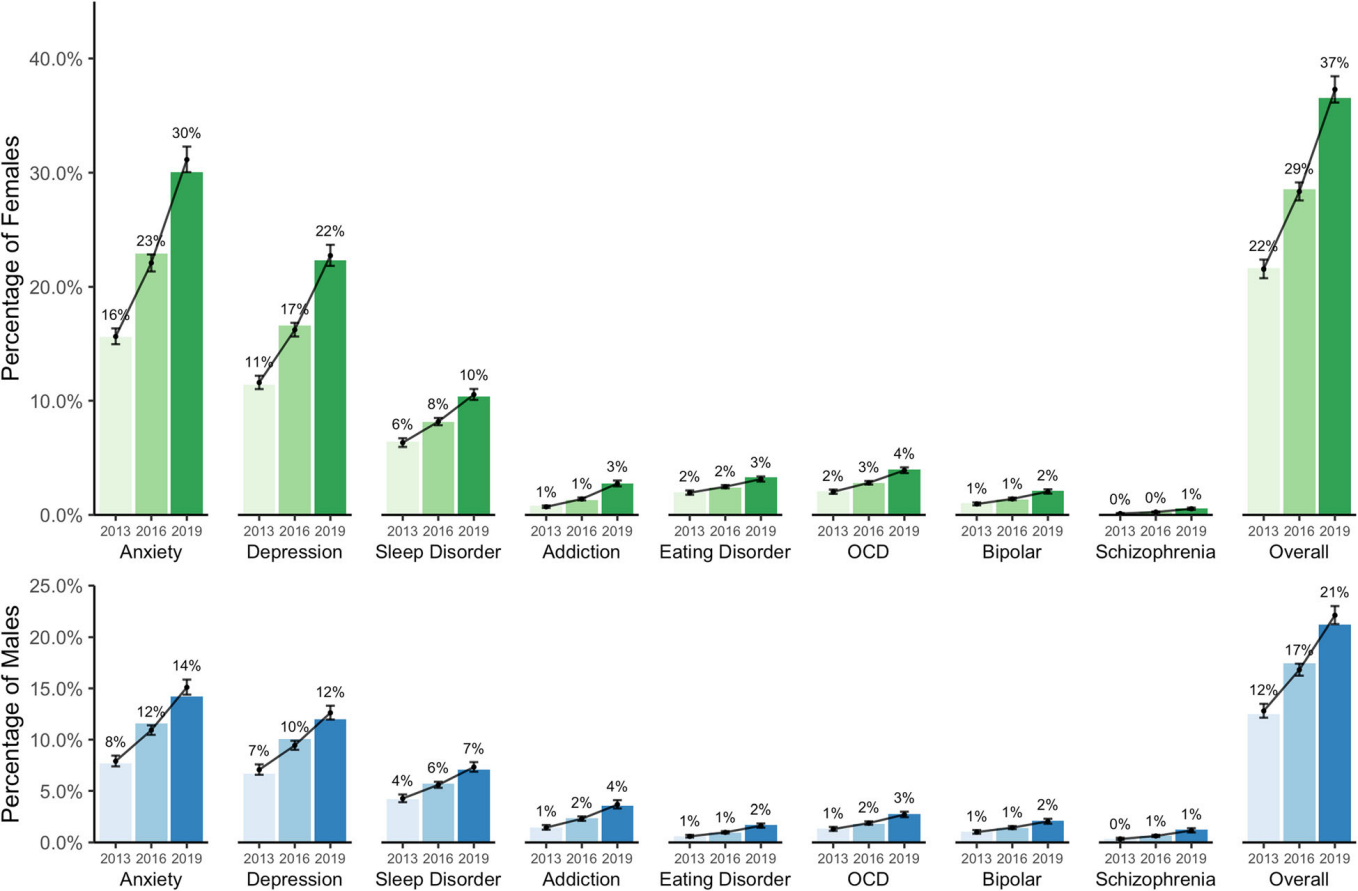
Proportion of students who received a diagnosis for a mental illness within the past 12 months, by sex

Figure 3 depicts the proportion of respondents who indicated that they had received a mental illness diagnosis from a medical professional within the past 12-month period. Statistically significant increases in proportions were observed across nearly all mental illnesses for both male and female students over time (see Additional file 1 for risk ratios and *p*-values). The largest increases were observed among diagnoses for anxiety and depression, with the proportion of female students reporting these diagnoses nearly doubling that of male students. Notably, modest increases were even observed in the proportions of students reporting having received diagnoses for much rarer mental illnesses, such as bipolar disorder, schizophrenia, and obsessive compulsive disorder.

**Fig. 4:**
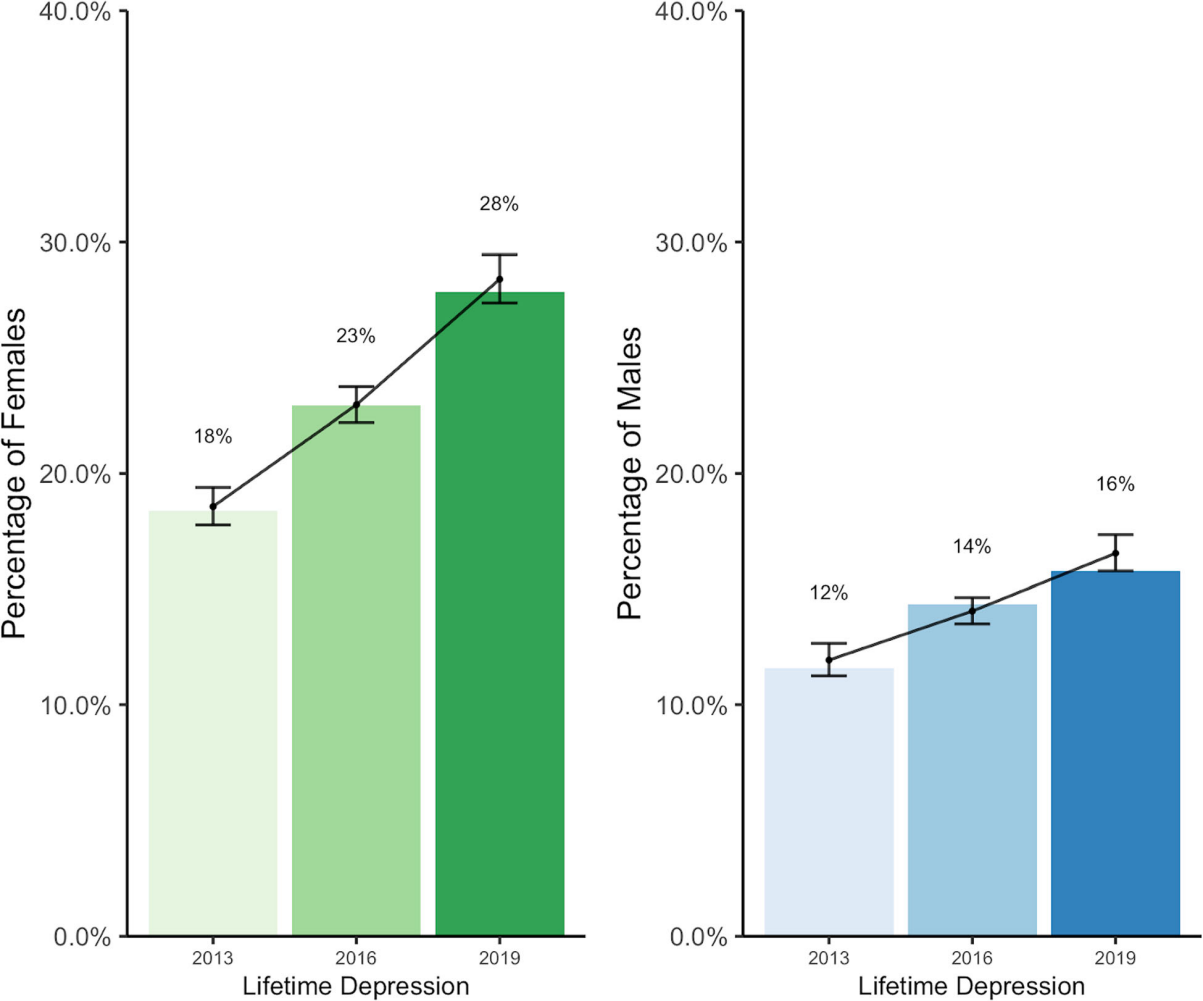
Proportion of students who received a diagnosis for depression in their life, by sex

In addition to past 12-month diagnoses, respondents were also asked whether they had been diagnosed with depression at any point in their life (Fig. 4). The proportion of students who reported ever having been diagnosed with depression increased over time (RR = 1.07, *p* < 0.001). This trend was observed among both male and female students, with a significantly larger proportion of female students reporting a diagnosis compared to males (RR = 1.55, *p* < 0.001). The annual relative risk increase for female students was RR = 1.07 (*p* < 0.001), compared to males (RR = 1.05, *p* < 0.001).

### Help seeking

**Fig. 5:**
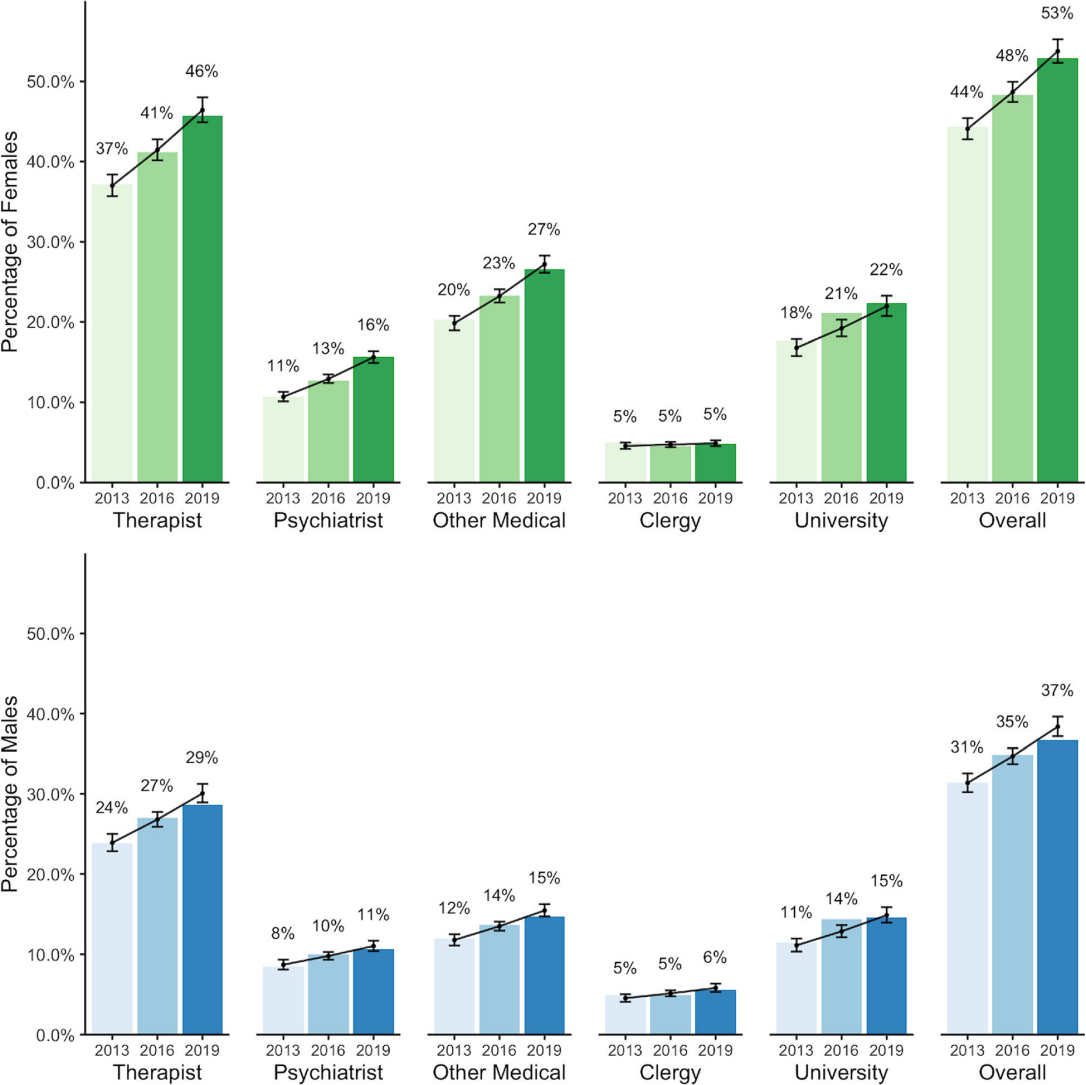
Proportion of students who reported seeking help for mental health related challenges from various sources, by sex

Respondents were asked whether they had sought help for a mental health-related problem from a number of sources over the past 12-month period (Fig. 5). The most frequent source students reported seeking help from was a counsellor, therapist or psychologist (46% of females and 26% of males in the 2019 sample). The most infrequent source utilized was a member of the clergy (e.g., minister, priest, rabbi, etc.), which remained relatively static with about 5% of participants reporting having sought help from this source across sexes and over time.

The proportion of students who reported receiving help from a counsellor, therapist, or psychologist increased significantly from 2013 to 2019 (RR = 1.04, *p* < 0.001). A significantly larger proportion of female students reported seeking help from this source compared to males (RR = 1.54, *p* < 0.001). Similarly, the proportion of students who reported receiving help from a psychiatrist increased significantly over time (RR = 1.06, *p* < 0.001), with a significantly larger proportion of female students reporting a diagnosis compared to males (RR = 1.22, *p* < 0.001). The annual relative risk increase was slightly higher for females (RR = 1.06, *p* < 0.001) compared to males (RR = 1.03, *p* < 0.001).

The proportion of students who reported receiving help from another medical provider (e.g., physician or nurse practitioner) increased significantly over time (RR = 1.04, *p* < 0.001), with a significantly larger proportion of female students reporting a diagnosis compared to males (RR = 1.68, *p* < 0.001). The annual relative risk increase for females was slightly higher (RR = 1.04, *p* < 0.001), compared to males (RR = 1.03, *p* < 0.001).

The proportion of students who reported receiving help from a member of the clergy (e.g., minister, priest, rabbi, etc.) was not significantly different by sex or year. Finally, the proportion of students who reported receiving help from their university or college mental health services increased significantly over time (RR = 1.04, *p* < 0.001), with a significantly larger proportion of female students reporting seeking help from student services compared to males (RR = 1.51, *p* < 0.001).

**Fig. 6:**
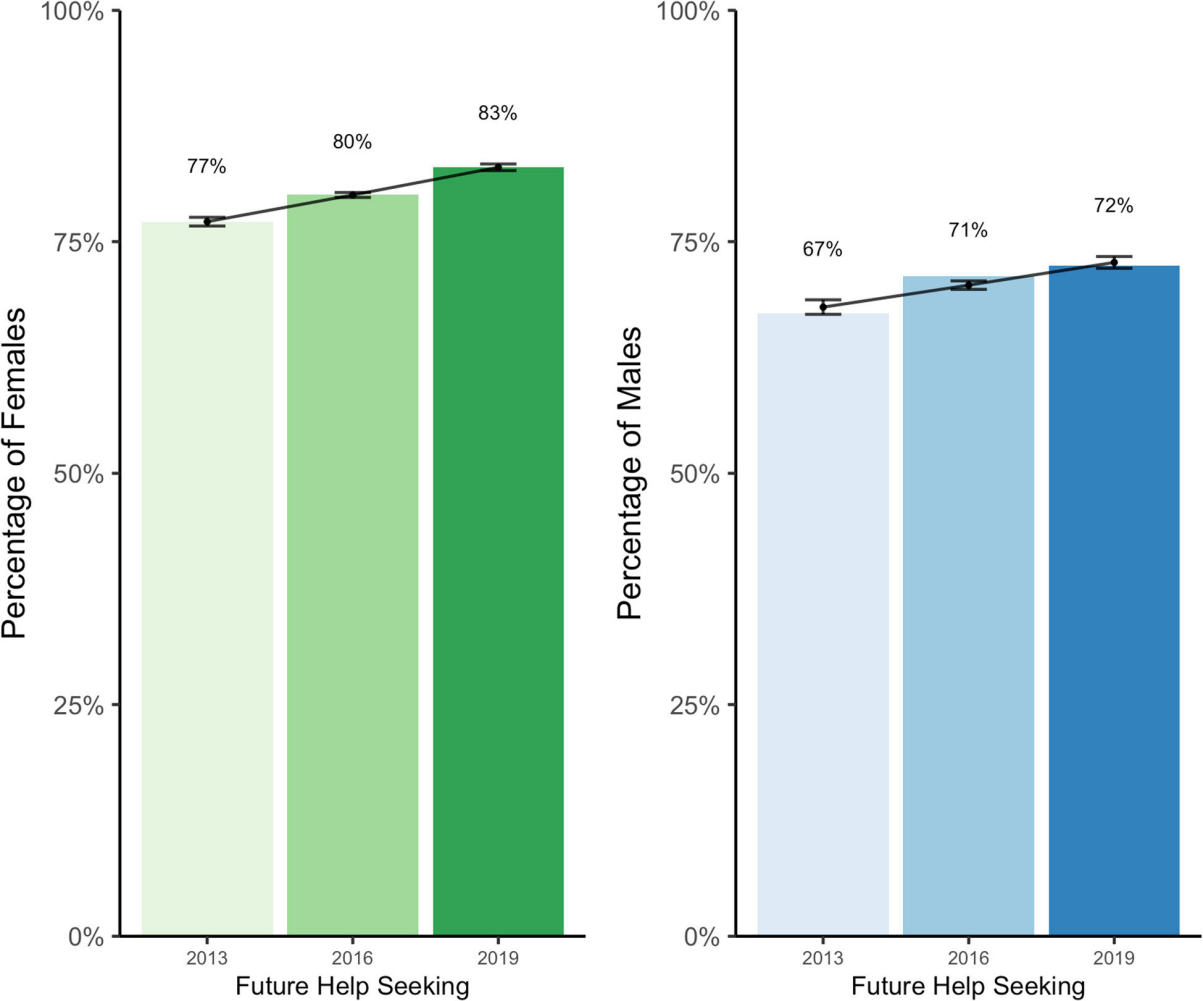
Proportion of students who reported intentions to seek help for mental health related challenges in the future over time, by sex

Finally, to gauge help seeking intentions moving forward, respondents were asked whether they would consider seeking help in the future should they experience a mental health problem (Fig. 6). The proportion of students who indicated they would seek help in the future increased significantly over time (RR = 1.012, *p* < 0.001), with a significantly larger proportion of female students reporting an intention to seek help in the future compared to males (RR = 1.13, *p* < 0.001).

## Discussion

Over the past decade, increasing attention has been paid to mental health issues among post-secondary students in Canada, with post-secondary institutions across the country reporting an increasing demand for mental health treatment services that they feel unable to meet. This increase in demand over the past several years has reportedly resulted in lengthy wait times, large caseloads, brief or limited sessions provided to clients, and a high rate of counsellor burnout [22–24]. Additionally, reports from counsellors suggest that the complexity and severity of students’ presenting issues have increased in recent years [25]. As a result, the question of whether or not a “mental health crisis” has emerged among this population has arisen [26, 27].

We examined multiple indicators for mental health and mental illness among Canadian post-secondary students comparing the data from the NCHA II cross-sectional surveys collected in 2013, 2016, and 2019. We determined whether prevalence estimates for mental health indicators had increased significantly over time, as well as whether the rate of change differed for male and female students. Our analysis revealed that the proportion of Canadian post-secondary students reporting above average stress, symptoms of psychological distress, diagnosed mental illnesses, and help seeking for mental health related challenges indeed significantly increased between the 2013 and 2019 iterations of the survey, with few exceptions. In nearly all cases, higher proportions were observed among female students compared to male students, though the rate of change was only significantly different between sexes for some indicators, indicating that the proportion of students self-reporting mental health challenges has increased at a similar rate for males and females in most cases.

Stress has long been reported among post-secondary students, with a well-established link between excessive stress and negative impacts on mental health [28, 29]. Additionally, associations between poor mental health and declines in academic performance have also been observed [15]. We found that mean stress levels reported by students increased significantly between 2013 and 2019, with female students reporting a higher mean stress level within the past 12 months compared to male students. Categorically, while the proportion of students reporting ‘average stress’ decreased, we observed increases in both the proportion reporting ‘tremendous stress’ and ‘no stress,’ with more male students reporting ‘no stress’ compared to females. This may suggest that while existing stress reduction interventions have resulted in some students improving, other students are getting worse. Recent research suggests that female students experience more frequent and severe stress compared to their male counterparts. Stress remains an imperative area of concern and the need for further investigation into the sources of stress among post-secondary students is clear. Tools such as the Post-Secondary Student Stressors Index (PSSI) [30, 31] have been created to do just this, and aim to enable post-secondary institutions to make evidence-informed decisions regarding the targeting and delivery of their upstream mental health services, including stress reduction programming.

Research among post-secondary students in the USA has revealed an association between common mental illnesses, such as anxiety and depression, and negative academic outcomes [15]. In this study, the proportion of students who reported having been professionally diagnosed with a mental illness increased significantly between 2013 and 2019, with a larger proportion of females reporting diagnoses than males. This finding is consistent with previous research and patterns of sex-based diagnosis in the broader population [32, 33]. Anxiety and depression remain the most common mental illness diagnoses among the student population, consistent with previous research, with the prevalence of these diagnoses appearing to increase over time. Whether or not the increases observed constitute a “true” increase in prevalence, however, is up for debate. It is possible that the observed increases in prevalence may stem from a combination of increased mental health awareness and a reduction in stigma, resulting in more students reporting and seeking help for mental health-related challenges. Another point of consideration is that increasingly, more students seem to be beginning their post-secondary educational careers with pre-existing diagnoses [34]. Therefore, the post-secondary environment is not necessarily creating an increase in the prevalence of mental illnesses, though it may be a contributing factor. Notably, recent research has shown that post-secondary students do not appear to have worse mental health than the general population, therefore challenging the notion of there being a “mental health crisis” among the Canadian post-secondary student population [35]. This is not to say that post-secondary mental health in Canada does not deserve attention, but suggests that mental health care for emerging adults both within and outside of the post-secondary institution must be bolstered.

Improved targeting and bolstering of upstream mental health services, in particular, may alleviate the current burden on downstream services (e.g., counselling, therapy) observed on post-secondary campuses [36] as well as in the general population [37, 38]. Upstream services may include mental health promotion activities and/or toolkits that assist students with bolstering and maintaining resilience in the face threats to one’s mental health. For example, “Surviving to Thriving,” a self-help resiliency toolkit designed for post-secondary students [39], may be a useful resource to distribute to incoming undergraduate students. This toolkit invites students to better understand changes in their mental health, and lay out a plan of action for how to effectively respond to common stressors within the post-secondary setting. Resilience has long been identified as an effective mediator of the relationship between stress and mental health outcomes, with individuals reporting higher levels of resiliency experiencing more positive mental health outcomes when compared to their low resiliency counterparts [40]. Students should also be educated about adaptive coping methods [41]. An oft-cited, maladaptive coping mechanism used by students to manage mental health challenges is substance use. Mental health education campaigns directed at students should emphasize healthier coping mechanisms, including developing strong social support networks and self-care regimens. In addition to the bolstering of upstream mental health services, improved connections between post-secondary institutions and community-based mental health care are warranted to ensure continuity of care beyond the academy [42].

Previous research has investigated mental health help seeking behaviours among the post-secondary student population, identifying a number of barriers including stigma [14, 43], lack of perceived need [44], and doubts surrounding treatment effectiveness [44]. Few studies have explored whether improvements have been observed in students’ willingness to seek help. Our analysis revealed the proportion of students seeking help significantly increased across all sources (with the exception of seeking help from members of the clergy) between 2013 and 2019, regardless of sex. A larger proportion of females than males reported seeking help from nearly all sources, which is consistent with previous research [14, 44, 45]. Increases were also observed in the proportion of students who expressed an intention to seek help in the future if necessary. This may provide support for a reduction in mental illness-related stigma and increase in overall mental health literacy among post-secondary student populations having occurred since 2013, both noted as important factors in the relationship between mental health help seeking attitudes and behaviours [46]. While a larger proportion of female students indicated an intention to seek help in the future, an increase in willingness was also observed among male students. This is notable, given that previous research has revealed a strong reluctance for help seeking among male students as a result of perceived stigma [12, 14, 47, 48]. One area with respect to help seeking on post-secondary campuses that has not been well explored to date is the perceived accessibility of mental health services from the student point of view. Actions have been taken within the broader post-secondary community to increase the availability and degree of services offered (i.e., the Okanagan Charter and the National Standard for Student Mental Health), we are not aware of any rigorous data available (particularly at a national level) regarding students’ perceptions of access to services and whether or not this has improved over time. This may present an interesting opportunity for future research regarding help seeking among this population.

### Limitations

The findings reported here are based on self-reported data which is subject to social desirability bias, particularly given the sensitivity of the topic. It is possible that some participants may have distorted their responses in order to abide by social convention or failed to report a mental illness diagnoses as a result of perceived stigma. Though the response rate for the NCHA II may seem fairly low (19–20%), it remains in line with previous survey-based research conducted among post-secondary student populations. Of note, since the sampling method and sample size used is left at the discretion of each participating institution, it is difficult to draw conclusions about student representativeness. It is possible that selection bias may be impacting the observed results. For example, students who elected to participate in the survey may be systematically different than those who did not. However, the direction of this bias is difficult to discern. While it is possible that students experiencing more severe stress might be more likely to complete a survey on this topic, the opposite may just as easily be true. For example, a student who is severely stressed as a result of managing multiple priorities may not choose to participate in a survey with no tangible benefit (no benefits or incentives were provided for participation).

With respect to measurement, many of the questions within the NCHA II are less than optimal. While a well-established and widely-used survey instrument, there is limited information available regarding the validity of the measures used on the NCHA II, and in particular, those related to mental health [49]. Validity and reliability analyses conducted on these measures to date include only principal components factor analysis and reliability analyses (Cronbach’s alpha), both of which provide modest internal structure evidence for the mental health measures used [50]. However, other types of validation evidence have not been evaluated, including response processes, relations to other variables (i.e., comparison to established mental health measures), and test consequences. In addition, while it is useful to capture students’ overall feelings of stress through the use of a global stress measure, the survey does not currently include a validated method of assessing specific sources of student stress. As stress as a key predictor of the development of mental health difficulties, and understanding the sources of student stress is key to adequately targeting mental health promotion efforts, this is an important gap to highlight. In addition to these challenges, several of the survey questions asked participants to recall how they felt over the past 12-month period. The length of this timeframe may have resulted in some degree of recall bias, resulting in misclassification.

Finally, no sample weights were applied to the NCHA II data, making it difficult to evaluate the representativeness of results to the broader Canadian post-secondary population. However, the age and sex breakdown of the sample is similar to that of the wider Canadian post-secondary population, according to Statistics Canada’s Post-Secondary Information System for the 2016 academic year [51].

## Conclusions

Our analysis indicated that the proportion of students self-reporting mental health related challenges, including stress, psychological distress, and diagnosed mental illnesses increased between the 2013, 2016 and 2019 iterations of the NCHA II conducted among Canadian post-secondary students. However, it is important to note that these results should be interpreted with caution. While we observed an upward trend in the data analyzed here, we cannot say whether this indicates an ongoing trend moving forward. We would be more confident in the results of a trend analysis conducted with more than three time points, particularly given the cross-sectional nature of the NCHA II data and its associated limitations. Overall, improved targeting and bolstering of upstream mental health services at the post-secondary level in addition to improved connections between post-secondary institutions and community-based mental health care are warranted.

## Supplementary Information

**Additional file 1: Table A-1.** Relative differences by year for male and female students and relative difference between male and female in 2019. This file contains Table A-1, which displays the risk ratios and 95% confidence intervals for each variable in our analysis. The table presents change per year for females, males, and the 2019 difference (males vs. females).

**Additional file 2: Table B-1.** Number of cases omitted by model outcome. This file contains Table B-1, which displays the cases omitted through the complete analysis approach taken to missing data in the datasets (Table requested by reviewer).

## Data Availability

The data used to conduct this study is available from the American College Health Association (www.acha.org/NCHA). This data is accessible for members of the ACHA through the completion of a request form available on the website noted above. Information on institutional and individual membership and associated costs is also available at the ACHA’s website. No original data was generated by the current study, which used a secondary analysis approach.

## Abbreviations

NCHA II: National College Health Assessment II
ACHA: American College Health Association
RR: Risk ratio

## Footnotes

1 Mean response rates were determined for each year by averaging response rates at each participating institution. Individual response rates per institution were calculated as the number of students who participated divided by the total sample size used (i.e., number of students invited to participate).

## References

[1] Durand-Bush N, McNeill K, Harding M, Dobransky J (2015). Investigating stress, psychological well-being, mental health functioning, and self-regulation capacity among university undergraduate students: is this population optimally functioning?. Can J Couns Psychother.

[2] Robinson AM, Jubenville TM, Renny K, Cairns SL (2016). Academic and mental health needs of students on a Canadian campus. Can J Couns Psychother.

[3] Gollust SE, Eisenberg D, Golberstein E (2008). Prevalence and correlates of self-injury among university students. J Am Coll Heal.

[4] Eisenberg D, Gollust SE, Golberstein E, Hefner JL (2007). Prevalence and correlates of depression, anxiety, and suicidality among university students. Am J Orthop.

[5] Patel V, Flisher AJ, Hetrick S, McGorry P (2007). Mental health of young people: a global public-health challenge. Lancet..

[6] Pearson C, Janz T, Ali J (2013). Mental and substance use disorders in Canada (Catalogue no. 82–624-X).

[7] Sunderland A, Findlay LC (2013). Perceived need for mental health care in Canada: results from the 2012 Canadian community health survey-mental health. Heal Reports.

[8] Lang JJ, Alam S, Cahill LE, Drucker AM, Gotay C, Kayibanda JF, Kozloff N, Mate KKV, Patten SB, Orpana HM (2018). Global burden of disease study trends for Canada from 1990 to 2016. Can Med Assoc J.

[9] Godin I, Kittel F, Coppieters Y, Siegrist J (2005). A prospective study of cumulative job stress in relation to mental health. BMC Public Health.

[10] Crompton S. What’s stressing the stressed? Main sources of stress among workers: Statistics Canada: Ottawa; 2015. Available from: http://www.statcan.gc.ca/pub/11-008-x/2011002/article/11562-eng.htm

[11] Auerbach RP, Mortier P, Bruffaerts R, Alonso J, Benjet C, Cuijpers P, Demyttenaere K, Ebert DD, Green JG, Hasking P, Murray E, Nock MK, Pinder-Amaker S, Sampson NA, Stein DJ, Vilagut G, Zaslavsky AM, Kessler RC, WHO WMH-ICS Collaborators (2018). WHO world mental health surveys international college student project: prevalence and distribution of mental disorders. J Abnorm Psychol.

[12] Rafal G, Gatto A, DeBate R (2018). Mental health literacy, stigma, and help-seeking behaviors among male college students. J Am Coll Heal.

[13] Golberstein E, Eisenberg D, Gollust SE (2008). Perceived stigma and mental health care seeking. Psychiatr Serv.

[14] Eisenberg D, Downs MF, Golberstein E, Zivin K (2009). Stigma and help seeking for mental health among college students. Med Care Res Rev.

[15] Eisenberg D, Golberstein E, Hunt JB (2009). Mental health and academic success in college. J Econ Anal Policy.

[16] Duffy A, Saunders KEA, Malhi GS, Patten S, Cipriani A, McNevin SH (2019). Mental health care for university students: a way forward?. Lancet Psychiatry.

[17] McLeod J, Uemura R, Rohrman S (2012). Adolescent mental health, behavior problems, and academic achievement. J Health Soc Behav.

[18] Faulkner G, Ramanathan S, Kwan M. Developing a coordinated Canadian post-secondary surveillance system: a Delphi survey to identify measurement priorities for the Canadian Campus Wellbeing Survey (CCWS). BMC Public Health. 2019;19(935):1–11. 10.1186/s12889-019-7255-6 PMC6624908 31296190

[19] American College Health Association. American College Health Association - National College Health Assessment II: Canadian reference Group data report spring 2016. Hanover: American College Health Association; 2016. Available from: http://www.acha-ncha.org/

[20] American College Health Association. American college health association - National College Health Assessment II: Canadian reference group data report spring 2019. Silver Spring: American College Health Association; 2019. Available from: www.acha.org

[21] American College Health Association. American college health association - National College Health Assessment II: Canadian reference group data report spring 2013. Hanover: American College Health Association; 2013. Available from: http://www.acha-ncha.org

[22] Ng P, Padjen M (2019). An overview of post-secondary mental health on campuses in Ontario: challenges and successes. Int J Ment Health Addict.

[23] Patterson P, Kline T. Report on post-secondary institutions as healthy settings: the pivotal role of student services, health and learning knowledge Centre. Victoria: Association of Canadian Community Colleges; 2008. Available from: https://www.accc.ca/

[24] Cairns SL, Massfeller HF, Deeth SC (2010). Why do postsecondary students seek counselling?. Can J Couns.

[25] The Coordinating Committee of Vice Presidents Students of Colleges Ontario. White paper on postsecondary student mental health. Toronto; 2015. Available from: https://tinyurl.com/ycswzxnc

[26] Evans TM, Bira L, Gastelum JB, Weiss LT, Vanderford NL (2018). Evidence for a mental health crisis in graduate education. Nat Biotechnol.

[27] Xiao H, Carney DM, Youn SJ, Janis RA, Castonguay LG, Hayes JA, Locke BD (2017). Are we in crisis? National mental health and treatment trends in college counseling centers. Psychol Serv.

[28] Lazarus RS (1993). From psychological stress to the emotions: a history of changing outlooks. Annu Rev Psychol.

[29] Pearlin LI, Menaghan EG, Lieberman MA, Mullan JT (1981). The stress process. J Health Soc Behav.

[30] Linden B, Stuart H (2019). Psychometric assessment of the post-secondary student stressors index (PSSI). BMC Public Health.

[31] Linden B, Boyes R, Stuart H. The post-secondary student stressors index: proof of concept and implications for use. J Am Coll Heal. 2020:1–9. 10.1080/07448481.2020.1754222. 10.1080/07448481.2020.1754222 32432984

[32] Jalnapurkar I, Allen M, Pigott T. Sex Differences in Anxiety Disorders: A Review The Burden of Pediatric Psychiatric Illness: A Family Perspective View project. J Psychiatry, Depress Anxiety. 2018;4(012):106–17.

[33] Labaka A, Goñi-Balentziaga O, Lebeña A, Pérez-Tejada J (2018). Biological sex differences in depression: a systematic review. Biol Res Nurs.

[34] MacKean G. Mental health and well-being in post-secondary education settings. Ottawa: Canadian Association of College and University Student Services; 2011. Available from: http://tiny.cc/s3e1lz

[35] Wiens K, Bhattarai A, Dores A, Pedram P, Williams JVA, Bulloch AGM, Patten SB (2020). Mental health among Canadian postsecondary students: a mental health crisis?. Can J Psychiatr.

[36] Max A, Waters R. Breaking down barriers: mental health and Canadian post-secondary students [internet]. Ottawa; 2018. Available from: https://tinyurl.com/y7ovsypc

[37] Loebach R, Ayoubzadeh S (2017). Wait times for psychiatric care in Ontario. UWOMJ..

[38] Coalition of Ontario Psychiatrists. Help Wanted. Toronto: Ontario needs psychiatrists. Coalition of Ontario Psychiatrists. 2018. Available from: https://www.eopa.ca/sites/default/uploads/files/Ontario%20Needs%20Psychiatrists%20FINAL%20-%20August%207%2C%202018.pdf [cited 2020 Sep 5]

[39] Linden B, Dorland A, Stuart H. From surviving to thriving: developing personal and academic resilience - pilot program evaluation report. Toronto: Canada Life; 2018. Available from: https://tinyurl.com/y78bwy7e

[40] Hartley MT (2011). Examining the relationships between resilience, mental health, and academic persistence in undergraduate college students. J Am Coll Heal.

[41] Taylor SE, Stanton AL (2007). Coping resources, coping processes, and mental health. Annu Rev Clin Psychol.

[42] College Student Alliance, Ontario Undergraduate Student Alliance, Colleges Ontario, Council of Ontario Universities. In it together: taking action on student mental health. Toronto; 2017. Available from: https://tinyurl.com/yac2gjod

[43] Gulliver A, Griffiths KM, Christensen H. Perceived barriers and facilitators to mental health help-seeking in young people: A systematic review. BMC Psychiatry. 2010;10(113):1-9. 10.1186/1471-244X-10-113 PMC3022639 21192795

[44] Eisenberg D, Golberstein E, Gollust S (2007). Help-seeking and access to mental health Care in a University Student Population. Med Care.

[45] Czyz E, Horwitz A, Eisenberg D, Kramer A, King C (2013). Self-reported barriers to professional help seeking among college students at elevated risk for suicide. J Am Coll Heal.

[46] Beatie BE, Stewart DW, Walker JR (2016). A moderator analysis of the relationship between mental health help-seeking attitudes and behaviours among young adults. Can J Couns Psychother.

[47] Pedersen ER, Paves AP (2014). Comparing perceived public stigma and personal stigma of mental health treatment seeking in a young adult sample. Psychiatry Res.

[48] Davies MCB, Frank J, Dochnahl A, Pickering T, Harrison B (2000). Identifying male college students’ perceived health needs, barriers to seeking help, and recommendations to help men adopt healthier lifestyles. J Am Coll Heal.

[49] Rahn RN, Pruitt B, Goodson P (2016). Utilization and limitations of the American college health Association’s National College Health Assessment instrument: a systematic review. J Am Coll Heal.

[50] American College Health Association. ACHA-NCHA-II Reliability and Validity Analyses. Hanover; 2013. Available from: https://www.acha.org/documents/NCHA/ACHA-NCHAII_RELIABILITY_AND_VALIDITY_ANALYSES.pdf [cited 2020 Sep 1]

[51] Statistics Canada. Postsecondary enrolments, by program type, age groups, registration status, and sex. Table 37-10-0015-01. 2017. Available from: https://www150.statcan.gc.ca/ [cited 2019 Jan 31]

