# Supplementary material for: Cross-sectional trend analysis of the NCHA II survey data on Canadian post-secondary student mental health and wellbeing from 2013 to 2019
Source: BMC Public Health. 2021 Mar 25;21:590. doi: 10.1186/s12889-021-10622-1 (PMC7992810; doi:10.1186/s12889-021-10622-1)
Supplement: Supplementary file 1 — Additional file 1: Table A-1. Relative differences by year for male and female students and relative difference between male and female in 2019. This file contains Table A-1, which displays the risk ratios and 95% confidence intervals for each variable in our analysis. The table presents change per year for females, males, and the 2019 difference (males vs. females). [file 12889_2021_10622_MOESM1_ESM.docx]

**Additional file 1 - Table 1. Relative differences by year for male and female students and relative difference between male and female in 2019**

|  |  | Change per Year (Female) | Change per Year (Male) | 2019 Difference  (Male vs. Female) |
| --- | --- | --- | --- | --- |
| Help Seeking | |  |  |  |
|  | Therapist | 1.039 (1.033, 1.044) | 1.039 (1.031, 1.047) | 0.647 (0.625, 0.668) |
|  | Psychologist | 1.066 (1.055, 1.077) | 1.04 (1.025, 1.055) | 0.816 (0.766, 0.870) |
|  | Other Medical | 1.054 (1.046, 1.062) | 1.046 (1.034, 1.059) | 0.595 (0.566, 0.626) |
|  | Clergy | 1.012 (0.995, 1.028) | 1.042 (1.021, 1.064) | 0.997 (0.910, 1.092) |
|  | University | 1.046 (1.037, 1.055) | 1.050 (1.037, 1.063) | 0.663 (0.630, 0.698) |
|  | Overall | 1.034 (1.029, 1.038) | 1.034 (1.027, 1.041) | 0.712 (0.693, 0.732) |
| Distress | |  |  |  |
|  | Hopeless | 1.030 (1.027, 1.033) | 1.022 (1.018, 1.027) | 0.842 (0.826, 0.859) |
|  | Overwhelmed | 0.998 (0.997, 0.999) | 0.995 (0.993, 0.996) | 0.865 (0.859, 0.872) |
|  | Exhausted | 1.002 (1.001, 1.003) | 0.998 (0.996, 1.000) | 0.879 (0.872, 0.886) |
|  | Lonely | 1.014 (1.012, 1.016) | 1.013 (1.010, 1.017) | 0.843 (0.829, 0.858) |
|  | Sad | 1.016 (1.014, 1.017) | 1.019 (1.016, 1.022) | 0.787 (0.775, 0.800) |
|  | Depressed | 1.059 (1.054, 1.063) | 1.041 (1.034, 1.047) | 0.854 (0.831, 0.878) |
|  | Anxious | 1.032 (1.030, 1.034) | 1.029 (1.025, 1.033) | 0.738 (0.724, 0.753) |
|  | Angry | 1.035 (1.031, 1.039) | 1.025 (1.019, 1.031) | 0.847 (0.826, 0.868) |
|  | Self Harm | 1.080 (1.068, 1.093) | 1.074 (1.054, 1.094) | 0.613 (0.563, 0.667) |
|  | Considered Suicide | 1.102 (1.091, 1.112) | 1.076 (1.062, 1.091) | 0.943 (0.887, 1.003) |
|  | Attempted Suicide | 1.132 (1.106, 1.159) | 1.149 (1.111, 1.189) | 0.823 (0.692, 0.980) |
| Mental Illnesses | |  |  |  |
|  | Eating Disorder | 1.083 (1.061, 1.105) | 1.184 (1.134, 1.236) | 0.304 (0.244, 0.378) |
|  | Anxiety | 1.122 (1.113, 1.130) | 1.114 (1.100, 1.129) | 0.505 (0.475, 0.536) |
|  | Depression | 1.119 (1.109, 1.129) | 1.102 (1.086, 1.118) | 0.609 (0.570, 0.651) |
|  | Bipolar | 1.135 (1.104, 1.166) | 1.125 (1.084, 1.167) | 1.056 (0.870, 1.282) |
|  | Sleep Disorder | 1.089 (1.076, 1.102) | 1.095 (1.074, 1.115) | 0.674 (0.617, 0.737) |
|  | OCD | 1.116 (1.095, 1.137) | 1.130 (1.095, 1.166) | 0.644 (0.550, 0.754) |
|  | Schizophrenia | 1.292 (1.213, 1.375) | 1.228 (1.157, 1.302) | 2.928 (1.996, 4.296) |
|  | Addiction | 1.254 (1.218, 1.290) | 1.168 (1.132, 1.205) | 2.047 (1.718, 2.439) |
|  | Overall | 1.096 (1.089, 1.103) | 1.095 (1.084, 1.107) | 0.594 (0.566, 0.623) |
