# Supplementary material for: Cross-sectional trend analysis of the NCHA II survey data on Canadian post-secondary student mental health and wellbeing from 2013 to 2019
Source: BMC Public Health. 2021 Mar 25;21:590. doi: 10.1186/s12889-021-10622-1 (PMC7992810; doi:10.1186/s12889-021-10622-1)
Supplement: Supplementary file 2 — Additional file 2: Table B-1. Number of cases omitted by model outcome. This file contains Table B-1, which displays the cases omitted through the complete analysis approach taken to missing data in the datasets (Table requested by reviewer). [file 12889_2021_10622_MOESM2_ESM.docx]

Additional file 2 – Table B-1. Number of Cases Omitted by Model Outcome

|  | Female | | | Male | | |
| --- | --- | --- | --- | --- | --- | --- |
| Outcome | 2013 | 2016 | 2019 | 2013 | 2016 | 2019 |
| Distress Scale |  |  |  |  |  |  |
| Felt things were hopeless | 151 | 138 | 206 | 65 | 71 | 103 |
| Felt Overwhelmed by all you had to do | 77 | 131 | 155 | 32 | 80 | 84 |
| Felt Exhausted (not from physical activity) | 83 | 143 | 142 | 54 | 73 | 85 |
| Felt very lonely | 79 | 196 | 202 | 33 | 115 | 118 |
| Felt very sad | 141 | 159 | 258 | 53 | 89 | 154 |
| Felt so depressed it was difficult to function | 104 | 167 | 215 | 48 | 96 | 110 |
| Felt overwhelming anxiety | 102 | 170 | 188 | 51 | 105 | 113 |
| Felt overwhelming anger | 202 | 222 | 251 | 92 | 120 | 134 |
| Intentionally injured yourself | 83 | 157 | 190 | 43 | 90 | 113 |
| Seriously considered suicide | 73 | 165 | 204 | 39 | 91 | 117 |
| Attempted suicide | 129 | 188 | 232 | 68 | 103 | 138 |
| Mental Illness |  |  |  |  |  |  |
| Eating Disorder | 180 | 268 | 646 | 77 | 145 | 292 |
| Anxiety | 215 | 342 | 586 | 96 | 211 | 345 |
| ADHD | 127 | 179 | 261 | 57 | 100 | 127 |
| Bipolar | 127 | 192 | 246 | 56 | 107 | 131 |
| Depression | 150 | 207 | 380 | 61 | 128 | 178 |
| Sleeping Disorder | 269 | 266 | 386 | 119 | 162 | 194 |
| OCD | 86 | 131 | 187 | 42 | 72 | 84 |
| Schizophrenia | 179 | 348 | 605 | 69 | 151 | 280 |
| Addiction | 223 | 227 | 330 | 77 | 126 | 139 |
| Any Diagnosed Mental Illness | 590 | 749 | 1379 | 264 | 411 | 788 |
| Depression (Ever) | 357 | 172 | 322 | 247 | 106 | 219 |
| Help Seeking |  |  |  |  |  |  |
| Counselor, Therapist, or Psychologist | 129 | 154 | 357 | 57 | 105 | 159 |
| Psychiatrist | 209 | 260 | 484 | 78 | 130 | 189 |
| Other Medical Provider | 195 | 219 | 321 | 81 | 136 | 134 |
| Minister, Priest, Rabbi, Clergy | 340 | 293 | 456 | 157 | 164 | 172 |
| University’s Couseling or Health Service | 134 | 165 | 155 | 70 | 106 | 94 |
| Future help seeking | 169 | 113 | 123 | 98 | 93 | 61 |
| Overall Stress Level | 81 | 60 | 76 | 41 | 50 | 45 |
